# Epilepsy in children and young people as a stigma throughout history: identifying cultural, religious and educational factors to design strategies for contemporary social inclusion

**DOI:** 10.3389/fneur.2026.1870071

**Published:** 2026-09-29

**Authors:** José Gómez-Galán, Cristina Lázaro-Pérez, David Cobos-Sanchiz

**Affiliations:** 1 Department of Education and Social Psychology, Pablo de Olavide University, Seville, Spain; 2 Observatory on Mediation and Social Intervention in Organizations, Complutense University of Madrid, Madrid, Spain; 3 Faculty of Social and Health Sciences, University of Murcia, Murcia, Spain

**Keywords:** adolescents, children, culture, education, epilepsy, history, religion, stigma

## Abstract

Epilepsy ranks among the most extensively stigmatized neurological conditions in recorded history, affecting an estimated 50 million people worldwide. This paper examines the historical transformation of stigma associated with epilepsy in children, adolescents, and young adults across cultural and chronological contexts ranging from Mesopotamian antiquity to the digital present. A qualitative design was employed in which a critical narrative review of the scholarly literature furnished the basis for a historical-critical analysis of primary sources. Medical texts, religious writings, and legal documents were treated as the core material wherever they survived from the period under study, while the secondary literature—chiefly peer-reviewed studies, together with monographs and institutional reports—retrieved from databases including JSTOR, PubMed, Scopus, and Web of Science, and selected according to explicit inclusion and exclusion criteria focused on the social, cultural, religious, and educational dimensions of epilepsy in younger populations, served to identify and contextualize those sources and to situate them within existing interpretation. As an example of the depth of engagement with primary sources, the article presents an interdisciplinary analysis of one of the earliest semiologically precise literary descriptions of a childhood epileptic seizure—found in Mk 9:14−27—through a methodological convergence that combines, among other fields, philological studies, the direct analysis of original-language sources, the critical examination of manuscripts from scholarly critical editions, the social sciences applied to the study of history, and contemporary epileptological semiology, a combination rarely attempted in this literature. Across the various historical periods examined, recurring patterns of stigmatization have been identified, grounded in supernatural etiological frameworks, moral attribution, and persistent misconceptions about the nature and transmissibility of the condition. A central and undertheorized finding is the systematic ambivalence in the social valence of epileptic stigma: seizures interpreted among the socially marginal as demonic possession or divine punishment were, by the same cultural logic, recast among the powerful and the spiritually elevated as marks of divine election or charismatic predestination. This status-contingent inversion of stigmatic valence recurs across cultures and epochs. A related finding is that ideologically grounded stigma—most starkly, the eugenic doctrines of the nineteenth and twentieth centuries—proved more socially destructive in certain historical contexts than stigma of religious origin, precisely because it purported to rest on scientific authority, undermining the assumption that neurological knowledge alone attenuates stigma, which is in reality shaped by a broader range of factors. The findings underscore that the persistence of stigma is attributable primarily to complex sociohistorical processes implicating power and ideology, deeply rooted cultural traditions, the instrumentalized use of religion, and educational exclusion. Understanding these processes from a historical perspective is the necessary foundation for designing culturally sensitive strategies to promote social inclusion and reduce discrimination against children and young people living with epilepsy.

## 1 Introduction

Globally, epilepsy ranks among the most prevalent neurological disorders and is estimated to affect some 50 million people (1). Despite notable medical and scientific advances that have led to a better understanding and treatment of this neurological disease—characterized by its tendency to cause epileptic seizures and by its neurobiological, cognitive, psychological, and social consequences—epilepsy continues to be shrouded in stigma that significantly impacts the quality of life of those who experience it (2–4). This phenomenon of stigmatization, which manifests as negative social attitudes that lead to discrimination and exclusion, has deep historical roots and persists in various forms to this day (5, 6).

The historical trajectory of epilepsy reveals how it has been interpreted through various cultural, religious, and medical lenses (7–9). From antiquity, when its manifestations—which go far beyond generalized tonic-clonic seizures, including a wide range of phenomena such as absences, focal seizures with or without impaired consciousness, or autonomic and sensory symptoms—were conceived as divine or demonic interventions, up until its gradual medicalization in the modern era, the narratives constructed around the condition have shaped its social perception and the lived experience of those affected (10). Even with advances in neuroscientific knowledge and the availability of increasingly effective treatments, stigma persists, manifesting itself in both overt and covert ways and limiting access to education, employment, and full social participation (11, 12).

The burden of stigma is particularly harmful during childhood and adolescence, formative stages in which discrimination can undermine young people's identity formation, self-esteem, and life prospects (13, 14). Various studies document how, in different sociocultural contexts, children and adolescents with epilepsy face educational barriers, rejection by their peers, and reduced involvement in community activities, all fueled by misconceptions about the condition (15–17). In many societies, these barriers are reinforced by cultural factors that associate epilepsy with mental disorders, intellectual disability, or even contagion, as well as by religious interpretations that link it to divine punishment or supernatural influences (18, 19).

The persistence of these prejudices, though perhaps less explicit than in the past, continues to lead to social exclusion (20). This can result in concealment of the condition, undermining self-confidence and psychological well-being and increasing vulnerability to anxiety and depression (21–23). Preconceived notions about the safety or capabilities of people with epilepsy remain a barrier to employment and retention in the workplace (24–26), and the same misconceptions—chiefly that the condition is contagious or reducible to a mental health problem—also shape encounters with the healthcare system itself, at times affecting the quality of care received (27, 28).

These questions are not purely academic. Knowing where a prejudice comes from—which institutions sustained it, which texts transmitted it, which interests it served at different historical moments—is a prior condition for dismantling it with any lasting effect. The history of epilepsy-related stigma is not background to the contemporary problem; it is part of the explanation for why that problem has proved so difficult to correct. Identifying the cultural, religious, and educational channels through which stigmatizing beliefs have been carried forward allows us to address not just misinformation in its immediate form but the social and psychological structures that keep it in circulation. The record of past attempts to mitigate stigma—and there have been serious attempts, some of which have had genuine effect—carries lessons that purely clinical approaches to the question have sometimes failed to absorb. There is also a specifically contemporary dimension that the existing literature has not always engaged with sufficient seriousness. Digital technologies are now the primary environment in which many children and young people construct their understanding of the world—which implies new methodological approaches to researching this new reality—(29, 30), including their understanding of conditions such as epilepsy. The ubiquity of screens and their visual content introduces risks that did not exist a generation ago (31, 32), above all for patients with photosensitive forms of the disease, whose daily exposure to potential triggers has multiplied (33, 34). Epilepsy is not, unlike some conditions that were far more prevalent in earlier periods, a declining problem. In specific respects, the social and medical challenges it poses are greater now than they were in the past, and the digital present belongs in any historical account of stigma. This study attempts to include it.

The study analyzes how the stigma surrounding epilepsy has evolved across historical periods and cultural contexts, focusing on children, adolescents, and young adults. Its purpose is to identify the factors—cultural, religious, and educational—that have sustained that stigma over time and, drawing on this historical analysis in conjunction with current evidence, to propose strategies for social inclusion. The work is addressed to specialists across several fields: education, the social sciences, the humanities, and the health sciences all bear directly on these questions, and researchers in each will find dimensions of the problem that their own disciplinary formation has tended to leave out of view. The interdisciplinary approach—integrating historical, clinical, and sociocultural perspectives—is not a preference so much as a necessity, since no single discipline has produced an adequate account of the phenomenon on its own. There have been serious studies of epilepsy in its historical setting, including the foundational work of Temkin (10), the contributions of Magiorkinis et al. (35, 36), Wolf (5), Panteliadis et al. (37), and Kaculini et al. (8), among others. None has concentrated specifically on childhood and adolescence—stages of development with their own characteristics, including those bearing on psychological formation and education, and with their own particular vulnerabilities to the harms that stigma inflicts. That is the gap this study addresses.

## 2 Theoretical framework and methodology

Stigma associated with epilepsy is not a fixed object with a single, continuous history. It has been constructed and reconstructed differently in different times and places, shaped throughout by cultural, religious, and educational forces that themselves changed with changing political and social conditions. The analysis draws, as the preceding discussion has indicated, on perspectives from the history of medicine, sociology, cultural anthropology, social psychology, and public health—not because interdisciplinarity is a virtue in itself, but because no single one of these perspectives is sufficient on its own to account for what the historical record actually shows. A qualitative design was adopted that combined a historical-critical analysis of the main sources with other methods from the humanities and social sciences. The primary basis of this design was the identification and interpretation of a heterogeneous set of primary and secondary sources, which made it possible to propose historically grounded responses to the stigma associated with epilepsy.

### 2.1 Conceptualization of stigma in pediatric and adolescent epilepsy

The theoretical analysis is based on the conceptual framework of stigma, initially formulated by Goffman (38), which can be understood as a discrediting attribute that undermines an individual's social identity. Link and Phelan (39) expanded this perspective, particularly regarding the structural dimension of stigma, which operates through interconnected processes of labeling, stereotyping, social separation, loss of status, and discrimination. In health research, this has produced a three-fold conceptualization: enacted stigma, meaning the discrimination actually experienced by the individual; perceived stigma, the awareness that negative social attitudes exist in one's environment; and internalized stigma, the process by which those attitudes come to be applied by stigmatized individuals to themselves (40). In the specific case of epilepsy, stigma lies primarily in the magnitude and extent of the social exclusion suffered by patients, who experience rejection based, above all, on misconceptions, fear, and limited knowledge of the condition (41, 42). It is within this framework that the experience of children and adolescents with epilepsy can be properly placed—not as an isolated phenomenon, but as a position within a broader spectrum of social exclusion that shapes, over time, both educational trajectories and psychosocial development.

### 2.2 Research approach and methods

Both primary and secondary sources were consulted. The distinction matters and warrants a word of explanation. Primary sources include medical texts, religious writings, legal documents, and other historical materials spanning from ancient Mesopotamia to the present century. Where the original languages were accessible, these were analyzed directly; where they were not, well-established critical editions and scholarly translations were used, and the limitations this imposes on interpretive confidence are acknowledged where relevant. Secondary sources—peer-reviewed studies, book chapters, and monographs—were retrieved from specialized academic databases, among them JSTOR, PubMed, Scopus, and Web of Science, using combinations of keywords such as “epilepsy,” “history,” “stigma,” “culture,” “religion,” “education,” and “social inclusion,” applied individually and in conjunction. These searches were extended, where the historical or cultural scope of a given inquiry required it, to incorporate period-specific and context-specific terminology. No date restrictions were imposed on the literature, given that the study ranges across several millennia; no language restrictions were applied beyond the practical limits of the research team's linguistic competence, though it must be acknowledged that the secondary literature consulted is predominantly in English and other major Western languages, a constraint that may influence the coverage of scholarship produced in other traditions.

The selection of sources was guided by explicit criteria, even if not by a formal algorithmic protocol. Sources were included when they addressed the social perception, stigmatization, or cultural and religious interpretation of epilepsy in any historical period or cultural context; when they focused specifically on children, adolescents, or young people; when they examined the role of cultural, educational, or religious factors in shaping the social experience of the condition; or when they made a substantive contribution to the theoretical framework of stigma as applied to health and disability more broadly. Sources were excluded when their scope was restricted to clinical, pharmacological, or neurological aspects of epilepsy without substantive engagement with psychosocial or historical dimensions; when they addressed epilepsy exclusively in adult populations without any connection to developmental, educational, or pediatric considerations; and when their methodological basis was insufficiently documented to permit even minimal critical appraisal.

The review of the secondary literature was not designed as a systematic review in the strict methodological sense. No bibliometric or scientometric procedures were applied, and no formal quality-appraisal instruments were used to assess the included studies. The approach is better characterized as a critical narrative review, a method with a well-established tradition in the social sciences and the history of medicine, concerned less with exhaustive enumeration than with conceptual synthesis, the development and refinement of theoretical positions, and the critical interpretation of existing literature toward analytical ends that quantitative synthesis would not serve (43, 44). The aim, throughout, was not merely to inventory what has been written but to use the existing scholarship both to identify relevant primary sources and to interpret the historical evidence in relation to the central questions of the research. The total corpus of sources cited includes, alongside the peer-reviewed secondary literature, primary historical sources, institutional reports from recognized health and educational authorities, and reference works whose function is documentary rather than analytical; this heterogeneity reflects the nature of the inquiry, which spans disciplines and historical periods, and required the development of a coherent method of source criticism.

Regarding the transparency and traceability of the source-identification process—given that this study is situated primarily within the methodological framework of historical research and adopts a critical narrative review for source identification—the searches in the databases identified above were conducted iteratively between 2023 and 2026. The keyword searches were supplemented by citation chaining based on the reference lists of key sources. As part of the heuristic phase of historical research, this involved beginning with several relevant works and examining their notes and bibliographies in successive stages in order to identify further secondary scholarship and primary sources (45, 46). The materials identified through these searches were then subjected to critical assessment in terms of provenance, context of production, relevance, reliability, and evidentiary value (47). This iterative procedure was deemed appropriate for identifying material that is widely scattered across historical periods and distinct cultural contexts and examined from a variety of disciplinary perspectives. Unlike a systematic review, it did not aim at comprehensive retrieval through a fully prespecified search, screening, and selection protocol, which would not have been appropriate for the historical and interpretive objectives pursued here. In other words, at this stage of the research, the aim was to construct a historically meaningful and analytically sufficient corpus with which to address the research questions and to support the subsequent interpretation of the evidence.

Primary sources were therefore treated as the core basis wherever relevant materials survived from the period under study—including medical treatises, legal codes, religious writings, and various forms of narrative evidence. Secondary scholarship was used to identify and contextualize primary sources, to compare existing interpretations, and to provide the historical and social setting required for their critical analysis. When a claim rested on a single primary source, or when the secondary literature concerning particular aspects of the problem—or specific historical periods—was limited, an effort was made to state this explicitly in the text. The evidentiary standard applied throughout was that generally associated with historical reasoning: convergence among independent sources whenever possible, together with explicit qualifications when the surviving record depended on a single source, on testimonies that were not demonstrably independent, or on indirect evidence from which only a tentative inference could be drawn (47, 48). The evidentiary basis is necessarily uneven across periods and sections, since the survival, accessibility, and independence of the historical record vary considerably. This unevenness constitutes a limitation of the available evidence and has been taken into account in the interpretation. Qualitative content analysis (49–51) was employed to identify recurring themes, conceptual patterns, and structural regularities across both primary and secondary sources. Systematic comparison across cultural, geographic, and historical contexts was a central analytical strategy, with particular attention to socioeconomic, religious, and political variables that modulate the form and intensity of stigma across different settings and populations. The historical interpretation developed from this material was then used to identify recurrent mechanisms of stigmatization and to inform the discussion of present-day educational, clinical, and social responses.

Section 7, which we have included as a special section because of its importance, requires specific methodological comment, since it presents a detailed case study of how a paradigmatic primary source may be analyzed for evidence of stigma affecting children and young people in antiquity. It employs tools that are not standard in the health sciences or in social-scientific studies of stigma: specifically, Koine Greek philology, New Testament textual criticism based on the major critical editions, and the social history of Second Temple Judaism. The case for their inclusion deserves to be made explicitly. The passage under analysis in that section—found in the Gospel of Mark, specifically in Mk 9:14−27—contains one of the earliest, and on close examination one of the most precise, literary descriptions of a childhood epileptic seizure to survive from the ancient world. Its evidentiary value for the history of stigma—for understanding how the condition was perceived, labeled, and socially managed in a first-century Near Eastern context, and how that perception reflected and reinforced the stigmatization of a child—depends, however, on knowing with some confidence what the text actually says. And that is not trivial. Several of the Greek verbs used in this passage carry a range of meanings across different contexts; one (τρίζω) is a hapax legomenon in the New Testament, appearing only here; and the textual tradition presents meaningful variant readings at key points. A careful translation that takes account of all this could resolve some of these ambiguities in ways that might reflect either the meaning the author intended to convey or the observational and conceptual assumptions underlying the description. Working directly with the Greek, and with the critical apparatus of the major manuscript traditions, allows one to recover a level of semiological specificity—and a corresponding level of social-contextual meaning—that would otherwise remain inaccessible. The philological and textual-critical apparatus used in Section 7 is not, therefore, an exercise in biblical scholarship for its own sake. It is the method by which a piece of historical evidence about the stigmatization of a child with epilepsy is made legible. The resulting phase-by-phase comparison between the Markan narrative and modern descriptions of ictal phenomenology, placed within the theological and social context of the text's early reception, shows the analytical value of this approach.

The interdisciplinary orientation of the study as a whole reflects a conviction, grounded in the existing literature and reinforced by this analysis, that stigma as a historical and social phenomenon cannot be adequately understood from within any single discipline. Throughout the article, knowledge is drawn from the history of medicine, the sociology of health, social psychology, cultural and religious studies, and pedagogy, in the belief that each perspective makes visible dimensions of the problem that others occlude. Taken together, these methods made it possible to establish a connection between historical data, social theory, and contemporary research on stigma related to epilepsy. The resulting interpretation furnished the basis for identifying persistent forms of exclusion and for considering their bearing on present-day educational, clinical, and social strategies.

## 3 Historical trajectories of the stigma of epilepsy

### 3.1 Ancient societies and civilizations

The earliest documented traces of epilepsy date back to Mesopotamian civilizations. Cuneiform tablets dating from around 2000 BCE describe the condition as *miqtu*, literally “the disease of falling” (52, 53). These accounts, found in the Babylonian medical compendium known as *Sakikku* (8, 54), reveal a conception of epilepsy deeply intertwined with divine punishment or demonic possession. The various clinical manifestations of seizures—not only generalized convulsions, but likely a broader spectrum of paroxysmal events—were interpreted within this supernatural framework, with different semiologies attributed to the influence of specific deities or malevolent spirits (55, 56). As a result, the therapeutic approach prioritized exorcisms and purification rituals over empirical medical interventions. However, it is also important to consider Stol's edition and translation of the preserved parts of the *Diagnostic Manual (SA.GIG)* (57) (Tablets XXVI, XXVII–XXVIII, XXIX, and XXX), which focus on epilepsy. These sections outline a potential pharmacopeia for the disease, mentioning deer antler, blood, fumigation, amulets, and jasper, a stone associated with the moon. This would be consistent with the links established in this important source between epilepsy, the calendar, and the moon, which, curiously, have endured to this day in many cultures (58).

It must be borne in mind that observation was very important in the Mesopotamian world, especially of the heavens. In the astrological divination they practiced, which had a strong social impact, it is possible to see that some practices were based on the determination of complex ephemerides—which they established thanks to their mastery of celestial mechanics—to which they ultimately gave a religious interpretation; we are faced with a paradigm of cultural synthesis between the rational and the magical (59, 60). Similarly, their understanding of epilepsy undoubtedly stemmed from a profound observation of the disease, which was also later interpreted—they always spoke, in all phenomena, of cause and effect—within mystical and supernatural frameworks. Proof of this is that they correctly specified, using a rich technical vocabulary, that it also affected animals and could occur from birth. Precisely in relation to childhood and youth, the focus of our interest, it should be noted that social reactions to children affected by this disease were significant (57). Stigma was already present in these remote historical moments. It was also interpreted, as was the case throughout the Near East, as a punishment from the gods or malevolent spirits against their parents. Since many of these entities dwelt in the heavens, we should not be surprised by a possible connection to celestial influence, which may have been the origin of this tradition that later became so widespread. However, as Fales argues (61), despite this magical-superstitious mindset, the Mesopotamians made an effort to classify the symptoms of the disease in detail, which, in turn, exerted a broad influence that extended from pre-Hippocratic Greek medicine to the medieval Jewish medical tradition.

In Ancient Egypt, we find a somewhat different perspective, though one that is also linked to magical thinking. Its medicine blended supernatural beliefs and anatomical observations, viewing illness as a disturbance of the body's vital forces (*ka*). Although there are no clear references in the important Ebers and London medical papyri (18th Dynasty, c. 1565–1300 BCE), the Edwin Smith Papyrus (c. 1700 BCE, although some of its sources may be much earlier, from the Old Kingdom, around 2600 BCE), which is surgical in nature, nevertheless offers one of the earliest descriptions of episodes consistent with epileptic seizures, although it would be linked to trauma (35, 62). Evidence can also be found in the Hearst Papyrus (c. 1500 BCE), which, in its treatment of this and other illnesses and of death itself, leads us to believe that the Egyptians considered its origin to be suprabiological, of a divine or demonic nature, for which they added magical formulas, incantations, and the use of amulets to the various remedies they applied (63), relying primarily on herbal remedies. What is significant is that this belief, which carries a strong stigma of superstition, remains prevalent today. A recent study found that in contemporary Egypt, there are still people with epilepsy who turn to traditional healers and that epilepsy continues to be understood as a form of possession (64). In the case of children, they were considered particularly vulnerable to these forces, with illnesses that caused seizures (65) or alterations in consciousness being most closely associated with possession by malevolent spirits. Although there is insufficient evidence to speak of the complete social exclusion of those who had epilepsy, it seems reasonable to assume that people with recurrent seizures would be stigmatized, in line with the Egyptian worldview that linked physical ailments to spiritual purity.

Similar cultural and religious frameworks, in which epilepsy was considered a disorder of supernatural origin, also prevailed in ancient times among Indian, Iranian, and Chinese civilizations (37). In the ancient Indian medical system, Ayurveda—initially transmitted through oral tradition and whose concepts are mentioned in the Vedas (between 3000 and 500 BCE), though with roots likely dating back much further (66)—it is defined as *Apasmara* (from *apa*, meaning negation or loss, and *smara*, memory or consciousness), and is described as the subjective sensation of sounds, darkness, or dreamlike states, possibly accompanied by falls, tremors in the hands, legs, and body, eye movements, teeth grinding, and foaming at the mouth (67). Therapies, which vary widely depending on the established nature of the condition, may range from the use of specific medications or adherence to particular diets to the recitation of mantras or the application of measures to awaken the channels of the heart and mind, which are blocked by the *doshas* (humors) (67, 68). In ancient China, the earliest document to mention epilepsy is the *Huang Di Nei Ching* (770–221 BCE), which focuses solely on generalized seizures and does not mention absence or partial seizures (69). Treatment was based on the principles of *Yin-Yang* and the *Wu Xing*, and included herbs, acupuncture, and massage. The sources we have been able to consult do not document the social treatment of epilepsy in these cultures with the specificity available for Mesopotamia or for the Greco-Roman world. What can be said is that the etiology recorded in the medical literature is of the same supernatural type found elsewhere, and that stigma of this kind is well attested in the same regions today (70, 71). Whether the one is continuous with the other we cannot demonstrate, and we do not assert it. The point is raised as a question the sources leave open, and one that bears directly on children and young people, who in these traditions as in others were the group least able to speak for themselves. In India, epilepsy is confused with a mental illness, and until recently, the law equated it with temporary insanity (71, 72). In traditional Chinese philosophy, which emphasizes the importance of demonstrating vitality in the face of adversity, a person with epilepsy who is not ashamed of it displayed such vitality (73), but if this were not the case, it implied that they would be socially stigmatized.

With varying interpretations, we find similar frameworks in other Eastern cultures. Although the foundations of Eastern thought, both philosophically and religiously, have developed metaphysical notions of immense depth and symbolism (74, 75)—as exemplified by the *Bhagavad Gita*—there is a marked divergence between religion and popular culture, rife with prejudices and stigmas rooted in superstitions. This phenomenon certainly occurs in all religions (76). The process of simplifying and reifying complex spiritual concepts results in an obscure law of moral causality that becomes a deterministic, fatalistic tool of social stratification. In the context described, and by way of example, a person's birth into a particular caste, or their suffering from a condition such as epilepsy, was no longer a cause for spiritual introspection, but rather a divine and inescapable destiny, an indicator of inherent purity or impurity. What, within the most sophisticated metaphysical frameworks of ancient India, could be interpreted as a manifestation of intense spiritual energy, a karmic echo of past lives, or even as a temporary dissolution of the ego—depending on the branches of Hinduism—drawing on mystical concepts of immense depth, when stripped of all philosophical subtlety, becomes a divine curse or a demonic possession, turning into a painful stigma that a person carries from childhood. This process, which creates a rift between transformative religious experience and prejudiced social application—in which authentic spiritual perceptions are distorted through institutional religious practice—has been present in one form or another in all religions, regardless of geographical location or historical period (77). This tension between esoteric and exoteric religious manifestations—perhaps stemming from a priestly interpretation (at that time serving the ruling elites) that favored social control over mystical exploration, the gradual textualization of oral traditions that lost their experiential context, and the political utility of the religiously rooted hierarchy in maintaining social order (78, 79), alongside the development of a mythology that facilitated and reinforced this transition—meant that the popular perception of epilepsy was no exception to the prejudices of this nature that arose in response to it.

In ancient Greece, with the emergence of Hippocratic medicine, there was a response to the prevailing supernatural interpretations. In his influential treatise *On the Sacred Disease* (c. 400 BCE), attributed to Hippocrates, it is argued that epilepsy was no more divine in nature than other ailments, but rather that its origin lay in a cerebral dysfunction resulting from humoral imbalances (10, 80). He asserted that the brain, and not supernatural entities, was the organ responsible for seizure activity. Despite this conceptual advance, the mystical connotations of epilepsy persisted in the Greek collective imagination, where those affected were often considered touched by the gods or, conversely, victims of a curse (81). Roman perceptions of epilepsy largely inherited Greek notions but intensified the stigma through the imposition of legal and social restrictions. The condition was frequently referred to as *morbus comitialis* (“disease of the assembly”), reflecting the belief that the occurrence of a seizure during a public gathering or religious ceremony constituted a bad omen that necessitated the suspension of the event, or similar interpretations in various other contexts, or as *morbus sacer* (“sacred disease”), which was often associated with demonic possession, madness, or divine punishment (9, 36, 82). There were significant legal restrictions on people with epilepsy regarding various activities (affecting their ability to marry, hold public office, or serve in the military), which highlighted a profound social marginalization that reinforced the view of this disease as a defect diminishing the individual's worth. In the case of children and young people, the associated stigma shaped their future prospects. In Greece, even with Hippocratic naturalistic explanations, childhood seizures were often interpreted as signs of supernatural intervention, which shaped their role in society (81). Similarly, in Roman society, children with epilepsy had restricted access to formal education, as beliefs about contagion, bad omens, and superstition led to them being viewed with fear and contributed to their educational exclusion.

Historical documentation of children and adolescents is, however, scarce. They do not typically appear as subjects describing their own condition, but rather as objects of another person's actions: a parent's request, a priest's ritual, a doctor's prognosis. This asymmetry is a characteristic of the records and is consistent with the secondary role that children played in those societies. In this context, a single well-documented case is more valuable than a general assertion. When sources allow us to view a child with epilepsy within the context of their own social environment, that case takes on decisive significance. That is why we have considered it essential to focus on one such case and explore it in depth, devoting a special section of this article (Section 7) to this purpose. The most significant case related to the historical period covered in this section is found in a passage from the Gospel of Mark (Mk 9:14−27), written in the first century CE, which contains what is very likely the most accurate description of a childhood epileptic seizure preserved from the ancient world. Furthermore—and this is what interests us most—it captures the social context surrounding the seizure: the crowd, the dispute among the scholars, the father who has already sought out every available healer, and the identification of the child by his symptom. Read using the tools of Koine Greek philology and New Testament textual criticism, the passage offers a step-by-step description of a generalized tonic-clonic seizure, an age of onset in early childhood, an indication of frequency, an account of injuries sustained from fire and water, and a postictal state that those present mistook for death. It also demonstrates, with a clarity unusual in any other ancient source, the mechanism by which a neurological episode became a moral and spiritual condition attributed to the child and his family. Given its enormous relevance and its decisive role as evidence in the overall analysis we are conducting, we examine it in depth and in its entirety, using the discipline-specific tools, in the dedicated section. It is a paradigmatic example of work with primary sources and must necessarily be presented separately from the chronological survey that comprises the rest of Section 3.

### 3.2 Ambivalence regarding stigma: modulating factors

Of course, from ancient times to the present day, stigma has not affected everyone or all segments of the population equally. It was mitigated—or even eliminated—when the person belonged to the ruling elite. On the contrary, suffering from epilepsy could, paradoxically, add a layer of mysticism that reinforced the image of someone predestined for greatness. One example is Julius Caesar, who, according to historical sources (Suetonius, Appian, and Plutarch, among others), suffered from headaches, seizures, and personality changes (83). A member of the powerful Roman patrician family, the *gens Julia*, he probably had epilepsy, migraine with aura, or some other cerebrovascular disorder (81, 84). This demonstrates that stigmas, beyond the actual consequences of the illnesses on which they are based, are heavily influenced by social contexts and prejudices, and that interpretations were—and are, since this is something that also occurs today, making this lesson from history of great value to us—highly ambivalent (85). Thus, what for people of low social standing who experienced seizures or trances was demonic possession, for those belonging to the elites was a sign of being in direct contact with the divine. This could have bestowed upon Caesar a sort of mystical aura or the status of being chosen by the gods, an interpretation he might even have fostered himself to consolidate his image as a charismatic leader with an exceptional destiny. Although sources suggest that he tried to conceal his seizures or sought a secluded place to recover, it is nonetheless likely that, in his private life or within a very small circle of trusted confidants, this reinforced his resilience in the face of adversity. This illness could have been a sign of his *virtus* (virtue, courage, manliness, excellence) according to the mindset of his time. His personal charisma, his immense political and military prowess, his control over public perception, and the vast power he had accumulated could have nullified or reinterpreted the superstitions prevalent at that time.

Throughout history, this has also been true of other figures. It is speculated that some of great prominence, such as Alexander the Great, Joan of Arc, or Napoleon, had epilepsy (86–88). And, as can be seen, this did not prevent a life marked by the admiration, respect, and devotion of their followers, demonstrating that stigma is subject to other factors of a social, economic, religious, educational, or political nature, and not solely to deep-rooted beliefs based on superstitions or traditions that single out those who are different (85, 89). In the context of our research, it is possible to conclude that this also applies today to children and young people from higher social classes, who are theoretically less affected by prejudice and stigma than those from lower social classes or those in disadvantaged social or geographic settings. Although few empirical studies have explored this issue in depth, some have, such as those by Newton and Garcia (90), Tiamkao et al. (91), Kirabira et al. (92), Ghaffar et al. (93), Rice et al. (94), and Hohmann et al. (95), among others. The studies by Ghaffar et al. and Hohmann et al., in particular, have found that socioeconomic position predicts internalized stigma and quality of life in samples drawn from high-income settings. The pattern is consistent across these works, although none was primarily designed to test this, and the results should be interpreted as indicative rather than as an established causal relationship; nevertheless, they are highly valuable for developing public policies aimed at addressing stigma.

### 3.3 Medieval perceptions

What the ancient civilizations bequeathed to later periods was not merely a set of beliefs but something more like a social grammar—a set of procedures for reading and responding to the visible symptoms of epilepsy. Subsequent developments did not simply replace those procedures. They were absorbed into them, modified in surface detail while retaining much of their underlying logic. Though particular cultures showed certain ambivalences, and some even a degree of reverence toward those who suffered seizures, as we have seen, the dominant social responses across the ancient world shared a common structure: fear, active exclusion, and in many legal systems the formal, institutionally endorsed sanctioning of discrimination. The shift toward a medicalized understanding was initiated, in the West, by Hippocratic and later Galenic theory—the first serious attempt to move the explanatory framework away from the supernatural and toward something resembling a physiological account (35, 55). But it was a slow move, and social behavior did not change at the same pace. For families with epileptic children, this meant that whether they lived in Mesopotamia, Rome, or the provinces of the early Christian world, the practical options were much the same: isolation, or recourse to religious healing in the absence of any effective medical alternative.

This was generally the case throughout the European Middle Ages. Understanding of epilepsy remained largely subject to theological doctrines and supernatural beliefs, although the rationalist legacy of Hippocrates also persisted. But the decline of the Greco-Roman scientific tradition in the West fostered a resurgence of mystical and religious interpretations, with the continued stigmatization of those suffering from the disease. Although there were some exceptions, as occurred in the Byzantine and Islamic worlds with scholars such as Ibn Sīnā—Avicenna (980–1037 CE)—who preserved and even expanded classical medical knowledge, attributing epilepsy to bodily pathological processes and even classifying different types of seizures and proposing early pharmacological treatments in works such as his *Canon of Medicine* (96, 97), in the medieval world the condition was not infrequently associated with demonic possession, relegating potential treatment to exorcisms and spiritual practices (36, 55, 98).

However, this cannot be generalized. Although it has been argued that the passage from the Gospel of Mark (9:17–27) examined below in Section 7, along with other references from Scripture or tradition, may have influenced the interpretation of epileptic seizures as cases of demonic possession (99, 100), and that this contributed to the stigmatization of the sick, certain clarifications and nuances must be made. First, Jesus's very attitude in the passage implies an invitation to overcome the stigma, as he shows compassion toward the affected person and rebukes the crowd's attitude, further advocating, after that encounter, for the person's integration, as reflected in the parallel passage in Luke 9:37–42 (85). Stigmatization and prejudice regarding epilepsy, throughout history, have extended beyond specifically religious frameworks, and the ambivalence surrounding the issue demonstrates that it was approached in various ways depending on diverse interests of different natures, ranging from social and political to economic. On the other hand, influential theologians such as Saint Augustine and Saint Thomas Aquinas did not deny the natural causes of epilepsy. It is considered, however, that medieval Christian theology consolidated the view of epilepsy as a primarily spiritual disorder (99), although it was recognized that, in reality, at its core, it was a physical pathology. What was said, however, was that demons or other supernatural entities were capable of manipulating bodily matter (the humors) to cause illness, and to do so they took particular advantage of the lunar phases, hence why those affected were also called lunatics. However, this explanation of the disease would be more closely linked to ancient pagan rites—consider, for example, lycanthropy, which was likewise associated with the influence of the Moon—than to specifically Christian ones, within the syncretic processes found in all religious phenomena.

As a result of all this, and although treatments were sometimes applied that were rational—originating from the Greco-Roman world, which some medieval scholars continued to employ—it was not uncommon that, consequently, based on the most superficial interpretations, many people affected by the disease, including children and adolescents, were excluded from social and religious life, subsequently barred from marriage, and, in some cases, subjected to severe practices such as forced fasting, flagellation, or confinement in monasteries. Saint Valentine was also invoked as a protector (101); his depictions in Christian art, accompanied by figures with epilepsy, have provided insights into the understanding of the disease at that time (102). Furthermore, it should not be overlooked that in most of the medieval West, the only institutions that took in, fed, and cared for the chronically ill were ecclesiastical: monastic infirmaries, cathedral hospitals, hospitaller orders, and, later, lay brotherhoods. A child with recurrent seizures whose family could not care for him was, in practice, more likely to be taken in by a religious house than by anyone else. In these places, the sick were fed, and records of them were even kept. Religious frameworks can involve both the stigmatizing etiology and the duty of care that mitigates it. Although this has been somewhat present throughout history, at that time in particular, the two were not considered to be in contradiction (103, 104).

The situation, however, worsened significantly during the witch hunts, which spanned from the late Middle Ages well into the Early Modern period (15th−18th centuries), particularly in central and northern Europe—coinciding with the religious wars that ravaged the continent—and had a particularly severe impact on adolescents and young women. Treatises such as the *Malleus Maleficarum* (1487) established an explicit connection between manifestations such as seizures, altered states of consciousness, or involuntary movements and witchcraft or demonic possession (101, 105–107). This placed people with epilepsy in a situation of extreme vulnerability, making them susceptible to accusations of witchcraft or of harboring demonic influences. Historical records from regions such as Germany and France document cases of adolescents with epileptic symptoms who were subjected to exorcisms, imprisoned, and even executed (105, 108–110).

Thus, for children and young people, this long historical period was marked by deep stigma, with interpretations rooted in ancient rituals, alongside instrumentalized interpretations within a divided and conflicted Christian religion—one deeply influenced by political interests—and with virtually no medical support. Childhood epilepsy came to be viewed as a sign of divine judgment or familial sin, leading to social isolation and, on more than a few occasions, persecution. The medical treatments available at the time were mostly ineffective, and people resorted to all manner of folk remedies, ranging from balancing the humors to various rituals, as was the case with many other pathologies or when facing devastating epidemics (111–114). Despite the intellectual progress that the Renaissance also brought to the medical field, public perception and the approach to epilepsy would not undergo a truly substantial change until the arrival of the scientific revolutions of the eighteenth and nineteenth centuries and, above all, the twentieth century. Even during these centuries, however, many earlier practices and interpretations persisted, perpetuating the stigma—from which the medical field itself was not exempt.

### 3.4 The modern period

The Modern Age (c. 1450–1790), which in the West spans roughly from the Renaissance to the Enlightenment, marked a period of progress in the understanding of epilepsy, despite profound contradictions (37, 115–117). Emerging scientific research began to undermine the supernatural explanations that had prevailed for centuries. However, social stigma and conceptual confusion proved particularly resistant, cruelly affecting the youngest members of society. During this period, epilepsy was frequently associated with notions of mental impairment, psychiatric illness, and deviation from the social order, leading to educational exclusion and, in many cases, institutionalization. Although the first attempts at specialized medical care emerged, these efforts tended to reinforce segregation rather than promote social integration.

The 16th and 17th centuries witnessed a gradual shift away from strictly religious interpretations toward more naturalistic hypotheses, particularly those that already attributed the origin of epilepsy to some dysfunction of the brain. Authors such as Paracelsus (1493–1541) explicitly rejected supernatural etiology and proposed that epilepsy stemmed from internal disturbances of the so-called vital spirits of the brain rather than from external forces. Although their theoretical framework remained tied to Renaissance medicine, these ideas helped shift the debate toward physiological explanations and paved the way for more mechanistic models that would influence later physicians, including Thomas Willis (1621–1675) (54, 118, 119). Paracelsus, albeit in a rudimentary manner, distinguished between forms of epilepsy with obvious convulsive manifestations and others without them, thereby recognizing a clinical diversity that anticipated, in an embryonic form, later classificatory developments. However, these theoretical advances did not automatically translate into improvements in the social or legal status of patients. Throughout much of Europe, exclusion from marriage, education, and employment persisted, justified by the belief that seizures revealed cognitive or moral inferiority. Medical discourse advanced slowly, while popular attitudes continued to marginalize those with epilepsy, especially children, whose opportunities for personal development and access to education were limited.

The dawn of the Modern Age also coincided with the establishment of asylums and institutions for people with neurological and mental disorders. Initially conceived as places of refuge, these centers often became instruments of social exclusion. Children with epilepsy, who in earlier times had been considered possessed, came to be classified as mentally deficient or incapable of learning, which served to justify their separation from the general education system. This process of institutionalization, observable in France, Germany, and England, set precedents for later developments in special education, although during the seventeenth and eighteenth centuries its predominant function was isolation rather than rehabilitation (120, 121). On the legal front, discrimination also persisted through the reinterpretation of regulations inherited from Roman law—such as the Code of Justinian—to restrict the civil rights of people with epilepsy.

Exclusion from formal education was a constant for children and adolescents with epilepsy throughout most of this period. Dominant medical theories, including humoral theories—which had already moved away from supernatural explanations—continued to view epilepsy as incompatible with full intellectual development. Many educators and physicians considered these young people unfit for regular school, fearing, moreover, that seizures would disrupt classroom activities or even that the disease was contagious (10, 65, 107). These prejudices erected barriers to access to education, even in elite institutions, where epilepsy could be interpreted as a sign of a hereditary defect. There were, however, some exceptions, such as the emergence of early forms of specialized education in some European countries that, although not intended solely for people with epilepsy, were oriented toward children and young people with special needs. These initiatives, however, tended to occur in segregated settings that, paradoxically, increased social isolation (36, 122). As early as the eighteenth century, the concept of moral treatment, based on structured routines and controlled environments, began to influence care for people with epilepsy. This approach represented an improvement over purely punitive methods, but proved insufficient to dismantle the deeply rooted social prejudices that continued to limit the life opportunities of those affected. At the same time, traditional and folk medicine—with herbal remedies, bloodletting, and amulets—persisted, especially in rural areas, contributing to the survival of non-scientific explanations and delaying the full acceptance of medical approaches based on empirical observation.

Overall, the modern period was marked by constant tension. On the one hand, the progressive medicalization of epilepsy fostered the development of brain-centered theories and the recognition of clinical diversity that included non-convulsive forms. On the other hand, social stigma remained firmly entrenched, manifesting itself in educational exclusion, the restriction of civil rights, and institutional confinement. For children and young people, epilepsy constituted a considerable obstacle to their full social integration. Although this era laid the foundations for modern epileptology, the true social inclusion of those affected did not begin to materialize until the nineteenth and twentieth centuries, with the advancement of systematic treatments and the progressive establishment of protective legal frameworks. This process, however, was very slow and uneven across countries.

### 3.5 Light and shadow in the early contemporary era

The 19th century and the first half of the 20th century constitute a decisive, yet deeply ambivalent, period in the history of epilepsy. There were notable advances in clinical neurology and in the classification of seizures, but social stigma not only persisted but, in various contexts, was intensified by the influence of eugenic theories. As noted earlier, prejudices against the disease cannot be limited to religious or cultural explanations; many other interests (of political, ideological, and economic origin, among others) bear equal or greater responsibility for segregation. The 19th century witnessed significant advances in neurological research, driven largely by the work of John Hughlings Jackson (1835–1911). Jackson formulated a fundamental definition of epilepsy as an occasional, sudden, excessive, and disordered discharge of nerve tissue, establishing the first coherent neurological model of ictogenesis (123, 124). His approach was pioneering in distinguishing between focal and generalized seizures and in linking epileptic activity to localized cortical dysfunctions, thereby laying the foundations for the localizationist approach in neurology, which was decisive for the subsequent development of epilepsy surgery. At the same time, clinicians such as Jean-Étienne Dominique Esquirol (1772–1840) and, most influentially, William Gowers (1845–1915), refined the clinical classification by popularizing the distinction between *petit mal* (absence seizures) and *grand mal* (generalized tonic-clonic seizures) (125–129). These advances represented a qualitative leap from the traditional notion of epilepsy as merely a “falling sickness.”

Alongside these medical advances, the nineteenth century saw the proliferation of specialized institutions for people with epilepsy, known as colonies or asylums. Prime examples were the Bethel Epileptic Colony in Germany and the Craig Colony for Epileptics in New York State, which opened in 1896 (130–132). Although they provided a certain degree of specialized care, these institutions functioned, in practice, as segregated communities, reinforcing the perception that people with epilepsy were unfit for ordinary social life (133). The education provided in these settings was limited, focused primarily on manual labor and discipline, and offered few opportunities for social reintegration, especially for children and adolescents.

In the late 19th and early 20th centuries, the emergence of eugenic doctrines, inspired by the ideas of Francis Galton, cast an even darker shadow over people with epilepsy. The condition was conceived as a hereditary defect that must be eradicated in the interest of racial improvement. This ideology gave rise to coercive policies in numerous countries (134–136). Although the extreme was, naturally, reached under Nazism, where people with epilepsy fell outside the prototype of the pure race and were included in its horrific eugenic policies, in other countries, such as the United States, more than twenty-five states passed laws allowing for the forced sterilization of people with epilepsy, and regulations prohibiting their marriage persisted well into the twentieth century (137, 138). These measures reflected a conception of epilepsy as an immutable genetic defect, ignoring its nature as a treatable neurological disorder. Children and young people occupied a particularly exposed position within this apparatus: sterilization statutes in the United States and, later, the compulsory institutionalization and, under National Socialism, the systematic killing of epileptic minors classified as hereditarily unfit, targeted individuals whose diagnosis was frequently established in infancy or early childhood, well before any capacity for consent or resistance could exist (129, 139). This is a paradigmatic example of how stigma can be far more prevalent in ideological or political contexts than in religious or cultural ones—although, naturally, all causes may be interconnected—even though the literature has traditionally focused on the latter as the primary motivating factors.

### 3.6 The paradoxes of the second half of the 20th century

Few periods in the history of epilepsy present a sharper contrast between clinical progress and social inertia than the decades following the Second World War. The medical transformation was real and substantial. Electroencephalography had entered standard clinical practice; new antiepileptic drugs were altering the prognosis for large numbers of patients; surgical techniques had been refined to a point that would have seemed implausible to the neurologists of the previous generation (36, 140, 141). None of this produced anything approaching the social inclusion of people with epilepsy. For children and adolescents, discrimination in education and social life continued much as it had before, persisting into the 1970s and, in many contexts, well beyond. Legal reforms were enacted in various countries, formally extending protections to those with the condition. But there is a considerable distance between legislation and altered social attitudes, and the gap between them did not close.

A key milestone of this period was the widespread adoption of the EEG in clinical practice. The first recordings of brain electrical activity, obtained by Hans Berger beginning in 1924 (his first paper appeared in 1929), were refined and became widespread in the 1950s. This technique offered, for the first time, an objective way to detect abnormal brain electrical activity, including between seizures (interictal activity). The EEG not only revolutionized the classification of epileptic syndromes and made it possible to distinguish epilepsy more precisely from other neurological or psychiatric disorders (142), but it also allowed for a deeper understanding of the different types of seizures, from absence seizures to focal or generalized seizures, which had a decisive influence on therapeutic strategy and neurophysiological research. At the same time, pharmacology advanced considerably. The emergence of antiepileptic drugs such as phenytoin (whose utility was discovered in 1938 and approved by the FDA in 1953), carbamazepine (synthesized in 1953, evaluated for epilepsy in 1963, and approved in 1965 in the United Kingdom), and valproate (1967) substantially improved seizure control in many patients, reducing their frequency and severity (143, 144). However, these medications were not without adverse effects, which sometimes included cognitive problems, sedation, or behavioral disturbances. Such effects could complicate social and educational integration, particularly in children, and fuel the erroneous association between epilepsy and intellectual disability that persisted in the minds of many parents and educators.

Surgery for epilepsy also underwent significant development. The pioneering work of Wilder Penfield in Montreal, who employed functional brain mapping, enabled precise localization and resection of epileptogenic foci in cases of drug-resistant focal epilepsy (145). Procedures such as temporal lobe resections or corpus callosotomies established themselves as viable therapeutic options, demonstrating that epilepsy did not have to be an intractable or degenerative condition. Access to these advanced interventions, however, was often limited by economic and geographic barriers, which exacerbated inequalities in care.

Despite this medical-scientific revolution, stigma and discrimination remained a daily reality for many people with epilepsy worldwide. In some places, legal restrictions on marriage even persisted (72, 146). In the educational sphere, segregation into special schools or the outright refusal of admission to mainstream schools remained common practices in various contexts. Bishop and Boag, in addition to their own research, reviewed a wide range of studies from different locations that revealed a lack of knowledge about epilepsy among teachers, ranging from misconceptions to limited first-aid knowledge for seizures (147). As late as the early 21st century, a study conducted in Taiwan found that a significant percentage of preschool teachers still believed that epileptic seizures were associated with insanity (148), which corresponded with studies from the second half of the twentieth century showing that teachers still had doubts about its contagiousness or its link to mental illness (149, 150)—ideas that remain present to a greater or lesser extent even in the current century (151–154) in places where stigma has a greater impact today. For their part, media representations often reinforced this stigma. Film and literature have tended to portray people with epilepsy—almost always focusing on the spectacular nature of tonic-clonic seizures—as violent, mentally unstable, or even possessed, thereby perpetuating historical stereotypes (155–158). Even within the medical community, it was not uncommon to find a psychiatric perspective on epilepsy, associating it with hysteria or other psychological disorders, despite growing evidence of its neurological basis (136, 159). Less conspicuous seizures, such as absence seizures or focal seizures without loss of consciousness, could be misinterpreted as inattention, daydreaming, or mere eccentricity, which gave rise to other types of prejudice.

It was also during the second half of the twentieth century that organized efforts to combat stigma gained significant momentum, led by patient and professional groups and accompanied by legal reforms. The founding of organizations such as the Epilepsy Foundation in the United States (1968) and the work of the International League Against Epilepsy proved decisive for public education, support for affected individuals, and political advocacy (160, 161). In countries such as the United Kingdom, Canada, and the United States, anti-discrimination legislation gradually removed legal barriers to marriage and ensured workplace protection. The Americans with Disabilities Act (ADA) of 1990 was a fundamental step forward in protecting against workplace discrimination in the United States, though social stigma proved more difficult to overcome. Globally, initiatives such as the *Out of the Shadows* campaign, launched jointly by the World Health Organization and the International League Against Epilepsy in 1997, sought to address stigma on a global scale (160, 162). However, the impact of these efforts has been uneven. Stigma remained particularly acute in low-resource countries, where supernatural or superstitious interpretations of epilepsy retained significant influence. By the end of the twentieth century, therefore, great strides had been made in understanding and treating epilepsy, but social attitudes had not kept pace. Children and adolescents with epilepsy remained particularly vulnerable to bullying, educational exclusion, and psychological distress. Although science defined epilepsy with increasing precision as a neurological disorder, the persistence of diverse cultural and historical prejudices meant that stigma continued to be a major barrier to full inclusion in social life.

### 3.7 The twenty-first century: stigma in the age of social media and screens

The 21st century has reshaped the landscape of stigma associated with epilepsy, introducing unprecedented dimensions, especially for children and adolescents. Digital platforms—ubiquitous and increasingly influential in shaping social interactions, public perceptions, and access to health information—present a double-edged sword for people with epilepsy. On the one hand, they offer valuable opportunities: online communities and telemedicine facilitate access to accurate information, peer support, and specialized medical care (163–165), and even enable the effective use of emerging artificial intelligence systems to improve the diagnosis and treatment of epilepsy (166). On the other hand, however, misinformation, stigma spread through social media, and cyberbullying act as powerful amplifiers of negative stereotypes, exacerbating the psychosocial distress of affected young people (167–171).

Bullying in the twenty-first century—and especially in this new digital context, cyberbullying—can take various forms, ranging from the misrepresentation of seizure videos—portraying them as possessions or using them in derogatory memes, depending on the geographical and cultural context—to direct mockery of any epileptic manifestation, whether convulsive or not. In general, although there are not many studies on the subject, it appears to be confirmed that children and adolescents with epilepsy, like others with chronic illnesses, face a significant risk of experiencing bullying, both physically and online (169, 170, 172–174). At the same time, traditional media portrayals, such as those in Hollywood films or television series, often continue to use visual tropes—in depictions of mental instability, violence, or supernatural phenomena—that can be associated with epileptic seizures, perpetuating a distorted image of the condition and contributing to public misunderstanding of the disease (175, 176). This can reinforce negative attitudes in the social and educational environments of young people. Meanwhile, the speed at which misinformation spreads online poses a formidable challenge for awareness campaigns (177–179). Social media algorithms, often designed to maximize engagement over accuracy, facilitate the dissemination of misleading narratives. A recent study analyzing social media posts about epilepsy found that a substantial portion contained inaccurate information (171). Because patients and their families use social media so widely, misleading information can be dangerous; hence the importance of training healthcare professionals in this area so they are aware of the topics circulating on these platforms (180). In general, there is no need for a deliberate disinformation campaign regarding epilepsy. The mere failure to provide accurate information can contribute to greater social isolation and delays in seeking appropriate treatment for children and young people. Equally important is the internalization of stigma within society itself.

A particular risk of the digital age affects people with photosensitive epilepsy. The proliferation of flashing lights and high-contrast visual patterns in video games, social media, and online advertising increases exposure to potential seizure triggers. This risk became internationally known following the mass incident caused by an episode of the *Pokémon* television series in Japan (181). Although regulations now exist requiring warnings, and there are numerous recommendations directed at designers of video games, films, television productions, web videos, and augmented or virtual reality images—as well as architects and designers of public spaces—to avoid visual stimuli capable of triggering seizures, and for governments and regulatory agencies to promote the filtering of potentially triggering media (182), the current situation remains far from ideal. Furthermore, seizures can be triggered not only by bright flashing patterns or repetitive flashes of color, but also by oscillating or static stripes, which can make the regulatory framework more complex. The truth is that visually provoked seizures (VPS) are both an individual and a public health problem that has emerged in recent decades, precisely due to the rise of the screen-based society, and which was unknown in the past.

Furthermore, paradoxically, the measures necessary to protect children with photosensitive epilepsy can, in and of themselves, give rise to new forms of stigmatization when applied indiscriminately or without adequate safeguards for participation. Video games, streaming videos, social media, concerts, school presentations, and other screen-related activities are now a substantial part of the social lives of children and adolescents. A child who must avoid certain content, leave an activity when flashing images appear, or rely on adults to determine whether a visual environment is safe may be singled out by peers as vulnerable or different. The resulting stigma does not necessarily take the form of overt hostility. It can arise through exclusion from shared leisure activities, restrictions imposed by families or schools, excessive displays of concern, or the child's own decision to hide their condition and withdraw from situations where a seizure might reveal it. Studies on children and adolescents with epilepsy have repeatedly linked such restrictions and the perception of being different to reduced participation, shame, concealment, and social isolation; the children themselves may report broader limitations than those recognized by their parents or teachers (183–186). Although these findings do not pertain solely to photosensitive epilepsy, the problem takes on a particular form in this group because the relevant triggers are embedded in everyday audiovisual environments and can occur without warning. Likewise, a lack of understanding persists in some sectors of society. For many people, seizures while playing video games are seen as a random occurrence without a causal link (187), and thus the condition is not taken seriously. The appropriate response, in all these scenarios, is not to isolate affected children from digital and cultural life, but rather to make these environments safer and offer reasonable alternatives without drawing unnecessary attention to the child in question (182). Otherwise, a measure intended to prevent seizures could reproduce a well-known mechanism of stigmatization: the shifting of responsibility from the environment that creates the risk to the individual who must avoid it.

However, digital technology has also opened new avenues for education and the defense of rights. Online platforms enable the large-scale sharing of personal experiences, raising awareness and humanizing patients' situations. Global campaigns such as Purple Day and initiatives led by organizations such as the International Bureau for Epilepsy (IBE) and the WHO have successfully used social media to disseminate reliable information and promote inclusion (188–190). In the educational sphere, anti-stigma initiatives are increasingly incorporated. Programs focused on peer education, teacher training, and first-aid training for seizures have proven effective in reducing prejudice and improving social integration in the school setting. However, the implementation of these strategies is far from uniform, and many regions still lack structured educational interventions to combat the prejudices associated with epilepsy effectively. Indeed, although medical advances continue to improve diagnosis and treatment, stigma remains a major psychosocial barrier, especially for young people. The digital age, with its inherent complexity, has introduced new forms of discrimination that require specific and coordinated responses across multiple spheres (media, technology, politics, the workplace, among others) to foster real and effective social inclusion for people with epilepsy. There are, however, three factors that have been present in stigma since the dawn of civilization: culture, religion, and education itself. And the lessons of history should enable us to refine and reorient strategies for change in these areas today.

## 4 Persistent factors in the shaping of stigma: culture, religion, and education

As we have seen, historical analysis reveals how certain interpretations and attitudes toward epilepsy have demonstrated a remarkable capacity for persistence, adapting and manifesting themselves through cultural, religious, and educational factors that continue to shape the experiences of children and young people today. Understanding these dynamics is the precondition for designing effective inclusion strategies.

### 4.1 The influence of culture: myths, language, and family dynamics

Cultural perceptions of epilepsy, often steeped in inherited misconceptions, remain a major source of stigma. Myths such as the contagious nature of the disease, which are completely unfounded from even the most basic rational perspective, persist in various communities (18, 149, 153, 191–196). To give an example, in Awaritefe's study, conducted in Nigeria (191), this was not only a widespread myth among the general population, but even medical students believed in it; in this context, they avoided eating, drinking, or sleeping in the same room as a person with epilepsy, nor did they touch them during a seizure. It is clear that this idea stems from the past but remains firmly entrenched in current beliefs. This demonstrates that factors such as a lack of education and training—particularly in the health sciences—are insufficient to explain this situation; the phenomenon of widespread superstitions, linked to the cultural notion of contagion, for example, undoubtedly contributes to their persistence within the collective. Moreover, it transcends even the African geographical context, where, as we have seen, more studies on the subject have been conducted. A study by Caprara (197), which examined the popular culture of Bahia (Brazil), demonstrated that a portion of the population considered epilepsy contagious and believed it was transmitted through the patient's saliva, which was regarded as a symbol of impurity. Also in Brazil, in the state of Paraíba, a study by Dantas et al. (198) found that the belief that epilepsy is contagious is present even among teachers. These are stigmas that endure over time.

Another common misconception is the association of epilepsy with mental health disorders or generalized intellectual deficits, a view still observed in developed countries (199, 200), and which contributes to further limiting educational and social opportunities. We are not saying that people with epilepsy, especially due to stigma, may develop mental health problems—a situation that is exacerbated and even leads to double stigma (201)—but rather that there is a belief that epilepsy itself is an exclusively psychological or psychiatric problem, not a neurological one. These perceptions are often reinforced by negative or inaccurate media portrayals, which tend to focus on the most dramatic manifestations (seizures) and associate them with danger or instability (175, 176). Even within the medical field, it is necessary to explore how stigma can harm the mental health of those with epilepsy. As Welch et al. (202) recently concluded, children and young people with epilepsy have a significantly higher risk of developing mental health problems compared to the general population; however, most of these problems are not recognized or adequately treated in clinical practice.

The very language used to refer to epilepsy can convey and perpetuate stigma. Historical terms such as “falling sickness” emphasize loss of control, and in many languages, there are expressions with negative connotations (10, 52). Promoting person-centered language (“person with epilepsy” rather than “epileptic”) has been shown to benefit self-perception and reduce stigma (203, 204). This eliminates labels that can single out the child or young person. Furthermore, family and community responses also vary by culture. While some foster strong family support, others tend toward overprotection or concealment, out of fear or shame, which restricts the child's social and educational participation. This overprotection, though well-intentioned, can lead to isolation, low self-esteem, and a delay in young people's acquisition of autonomy (205–207). Overcoming cultural stigma generally requires specific educational interventions, the promotion of responsible media portrayals, and public health strategies tailored to the community's cultural context, where the disease may be viewed in very different ways, which naturally calls for different actions.

### 4.2 The influence of religion: between superstition and compassionate support

Historically, as has been demonstrated, religious beliefs have exerted a powerful influence on the social perception of epilepsy. That influence has run in both directions rather than in one alone: the same doctrinal frameworks that have furnished vocabularies of possession and punishment have also, depending on context, supplied grounds for compassion, care, and communal support, and the balance between the two has depended less on the content of the belief system itself than on how it was locally interpreted and by whom. What follows traces this oscillation between the stigmatization associated with superstition and compassionate acceptance. Many traditions have reinforced supernatural interpretations, linking seizures (particularly those involving altered consciousness or automatic behaviors) to demonic possession, divine punishment, or spiritual impurity. These ideas, present in historical religious texts and certain traditions, persist today in various geographical regions (208, 209), especially in contexts where religious authority—and above all, with much greater intensity, in settings where fanaticism is present—takes precedence over medical authority. This religiously based stigma can have direct consequences for health, such as delays in seeking appropriate medical treatment. Studies in various regions show that many families first turn to spiritual interventions (prayer, exorcisms, traditional healers) before consulting a doctor, or continue to seek out healers even while receiving medical care (7, 210). This can prolong the time without an effective diagnosis or treatment, or lead to the mixing of incompatible therapies, which increases the risk of uncontrolled seizures and complications. Even in secularized societies, beliefs may persist that faith alone can cure epilepsy (211).

However, religious communities can also play a positive role. Many organizations have actively worked to combat stigma by integrating health education into their activities. The involvement of religious leaders who recognize epilepsy as a neurological disorder can be highly influential in changing perceptions and promoting adherence to medical treatment. Furthermore, faith-based health initiatives have provided crucial resources and support to underserved communities (212). Christian social reform has also been significant in the care of people with epilepsy and in the role of religion in coping with this condition (8). This historical ambivalence, as we have seen, is also reflected in the veneration of patron saints (such as Saint Valentine) or in the existence of healing shrines, which offered help but reinforced the idea that divine intervention was necessary (213). As discussed earlier, religious traditions are not divided into those that stigmatize epilepsy and those that support people with the condition. The same religion can encourage both approaches, for interrelated reasons. Which of the two prevails in a given community is determined by factors that are not in themselves religious: the availability and credibility of medical care, the education of local religious authorities, and the political uses to which the tradition is put at that time, among others. For this reason, the outcomes of interventions targeting religious communities are uneven, especially if not all factors are taken into account.

Furthermore, there has also been a well-established tradition linking religiosity to epilepsy, with various hypotheses attempting to explain it as a result of the person's social isolation, their need for religious comfort, or the experiences encountered during the course of an epileptic aura and the subsequent state of confusion (214), or in cases linked to ictal, postictal, or interictal phenomena. Particularly striking are the accounts involving children and young people, and all those episodes in which it was determined that seizures could alter the religious experience, either diminishing or enhancing it—even leading to conversions—especially in groups of patients with temporal lobe epilepsy (215–218). It is noteworthy that there are descriptions similar to those reported in near-death experiences (NDEs), so further research addressing both phenomena could be of interest, as many questions remain regarding their possible connection (219–224). It has also been suggested that historical figures with religious affiliations, such as Saint Paul or Joan of Arc, and especially mystics such as Saint John of the Cross or Saint Teresa of Ávila, may have suffered from epilepsy; however, some authors, such as Bone and Dein (212), question whether the connection between religiosity, mysticism, or religious conversion and this disease has been fully demonstrated.

Nevertheless, beyond the medical realm, it is most important to note that, in certain contexts, religion may also suggest that those who suffer from it might be chosen by the divine. Just as we saw that, in the case of the powerful, it was understood very differently from how it was among the common people—and especially among the poor and marginalized—we find the same dynamic in religious or spiritual spheres. Therefore, addressing stigma in children and young people today from this perspective requires significant collaboration among healthcare and education professionals, as well as community and religious leaders, who make decisions following a thorough analysis of the patient's situation. This holds true regardless of geographical or cultural context, though in some cases it would certainly be essential.

### 4.3 Educational barriers: misinformation, lack of support, and exclusion

The educational environment is a critical setting in which stigma and lack of knowledge create significant barriers for children and adolescents with epilepsy. Despite improvements in seizure control and inclusion policies, educational discrimination persists (225, 226). Factors such as the unpredictability of some seizures, the potential side effects of medication, or the neurocognitive effects associated with certain epileptic syndromes, combined with social stigma, are linked to academic difficulties, absenteeism, and poorer future educational prospects (227, 228). Furthermore, in certain contexts, the misconception that epilepsy is intrinsically linked to intellectual disability persists today. While it is true that specific epileptic syndromes can affect cognitive functions such as memory, attention, or executive function, the majority of young people with epilepsy have overall cognitive functioning within the normative range, although the risk of cognitive comorbidities is proportionally higher than in the general population (229, 230). However, existing biases in some settings may lead teachers and administrators to impose unnecessary academic restrictions, favor overly restrictive educational approaches, or maintain low expectations. Yet epilepsy is a disorder that can affect each child differently. In some cases, its impact on education is minimal, while in others it may require significant support within the school setting (225).

Another significant obstacle is the lack of specific teacher training. Many educators, in both high- and low-income countries, are unaware of basic aspects of first aid during seizures, triggers, safety measures, or the actual impact on learning. This lack of preparation can lead to fearful attitudes or restrictive decisions, such as limiting participation in certain school activities (231–233). Likewise, a lack of knowledge about how to recognize and manage non-convulsive seizures (e.g., absences) can lead to misinterpreting them as inattention or misbehavior. Bullying and social exclusion by peers are also serious problems. Young people with epilepsy experience higher rates of bullying, often because their peers misinterpret seizures—convulsive or otherwise—as a sign of weakness, oddity, or instability (172, 226). This hostile environment can lead to anxiety, depression, and a rejection of school, which negatively impacts performance and well-being. Although inclusive education policies exist that provide for accommodations, such as individualized education plans or seizure action plans, their implementation is often inconsistent, and many schools still lack trained staff, adequate supports, or clear guidelines. Teacher training and student awareness programs have proven effective in improving knowledge about epilepsy, increasing confidence in seizure management, and reducing stigma (232, 234), but their widespread implementation remains a challenge, especially in settings with limited resources or strong cultural stigma. Addressing these disparities requires coordinated interventions in teacher training, student awareness, and the strengthening of inclusion policies.

## 5 Impact of stigma across the lifespan of children and youth

Documenting that stigma harms children and young people with epilepsy is not, in itself, a difficult task—the literature on this point is extensive, and several of the studies cited throughout this article testify to it with varying degrees of methodological rigor. The more demanding question is whether the patterns of harm follow any systematic logic, and whether that logic connects to the historical processes traced in the preceding sections. The answer, we argue, is yes, and on two counts. First, the mechanisms by which stigma is transmitted to children and adolescents in contemporary societies are structurally continuous with historical mechanisms, even where the specific content has changed. The association of epilepsy with supernatural causation, which we traced from Mesopotamian cuneiform to early modern demonological literature, persists today in specific cultural and geographic contexts; but it is complemented, in other settings, by equally stigmatizing associations with mental illness, intellectual deficit, and social dangerousness—associations that are themselves historically produced, as Sections 3.4 and 3.5 showed. The form changes; the mechanism does not. Second, the developmental trajectory of childhood and adolescence makes the cumulative impact of stigma qualitatively different from its impact in adult life. Identity formation, peer integration, educational trajectory, and the construction of self-efficacy are all processes that occur during these years, and all are vulnerable to disruption by the sustained social devaluation that stigma represents. The damage done during these stages is not simply present suffering; it has long-term effects on life outcomes that extend well beyond the period of exposure.

What follows traces these dynamics across the major domains of young people's lives—educational, social, and psychological—with that structural argument as its organizing thread. In children, adolescents, and young adults, the stigma associated with epilepsy does not present uniform characteristics but rather interacts dynamically with the tasks and sensitivities specific to each stage of development, from early childhood through the transition to adulthood. Throughout history, we have seen evidence of this fact, and much of it remains today, particularly regarding the factors we have focused on. What is undoubtedly true is that its effects can manifest cumulatively and impact the development of self-concept, social integration, and life opportunities in multiple ways (13, 235, 236).

### 5.1 Early childhood (0–8 years): laying the foundation for self-concept

The early years of life are decisive in laying the foundation of self-concept, and early experiences related to the condition and the environment's reactions can have a profound and lasting impact. Perceived stigma in children with epilepsy is associated with lower self-esteem, poorer self-concept, and greater psychosocial difficulties, which can lead to social withdrawal (237–239). Family dynamics play a central role. The way the family responds to epilepsy—whether with open communication and acceptance, or with shame, avoidance, and overprotection—has a decisive influence on how the child integrates the condition into their emerging identity (240–242). In general, the experience of stigma at this stage can hinder emotional regulation, the development of social skills, and problem-solving, and increase the risk of mental health issues such as anxiety and depression, although a supportive family and community environment can act as a protective factor. The nature of the seizures—visible or not—and their potential cognitive or behavioral impact can also shape the environment's reactions and, consequently, the child's development (242, 243).

### 5.2 Middle childhood (9–12 years): peer perception and social identity

During middle childhood, the focus of social development shifts toward peer relationships, which become a fundamental mirror for the construction of self-concept. The literature on epilepsy, stigma, self-concept, and school life shows that this stage is particularly sensitive to peer evaluation (238, 244). Stigma, whether overt—teasing, exclusion—or subtle—avoidance, prejudice—and regardless of geographic context, can have a particularly painful impact, leading to feelings of isolation and low self-esteem (245–248). The visibility or invisibility of seizures also influences the reactions of those around them, which can generate different forms of social and school-based stigmatization. The academic sphere is also affected. Children with epilepsy may begin to internalize the idea that they have academic limitations, even when some of those limitations are perceived or amplified by those around them. Therefore, active support for the student is necessary to mitigate these negative perceptions; otherwise, a stigmatizing environment reinforces them (205, 249).

### 5.3 Adolescence (13–18 years): autonomy and the desire for normality

Adolescence intensifies these challenges as the task of forging an independent identity coincides with the need to manage a chronic condition such as epilepsy. This stage can be particularly affected by stigma, which hinders important milestones such as the onset of romantic relationships, planning for educational or professional futures, and the development of skills for independent living (13, 235, 236, 241). A characteristic tension arises between not being singled out because of the condition and the demands of managing epilepsy, including medication, restrictions, and the possibility of new seizures. This tension may be associated with concealing the diagnosis, coping difficulties, and avoiding situations in which the condition might become visible, which typically triggers anxiety and significant mental health problems (250–252). Fear of rejection can lead to concealing the condition, a phenomenon consistent with the notions of managing stigmatized identity and courtesy stigma described by Goffman (38). In pediatric and adolescent epilepsy, the literature on disclosing the diagnosis confirms that perceived stigma constitutes a consistent barrier to communicating the condition to peers and other significant others, further limiting the opportunities for social, vocational, and relational exploration typical of this stage. As a result, depressive disorders are not uncommon in adolescents with epilepsy (239, 253–255).

A related and still under-published problem, though far from rare in specialized epilepsy centers, deserves mention here: the coexistence, in the same patient, of epileptic seizures and psychogenic non-epileptic seizures (PNES) (256). Adolescents who present with both disorders carry a distinctive burden, living with the constant uncertainty of not knowing, from one episode to the next, whether a given seizure is epileptic or psychogenic in origin, and this uncertainty compounds the diagnostic difficulty already inherent to the dual presentation. The condition typically brings with it a considerable emotional toll and a recurring back-and-forth between anti-seizure medication and psychological or psychopharmacological treatment. This pattern fits uneasily with the adolescent's own need for a settled, singular narrative about what is wrong and what will help.

### 5.4 Transition to young adulthood (19–25 years): independence and structural barriers

The transition to adulthood marks another critical period, in which the quest for independence often clashes with persistent institutional and social barriers. There is consistent evidence, as demonstrated by a study by Roessler et al. (257), of employment discrimination and structural obstacles for young people with epilepsy, particularly regarding access to and retention of employment. Internalized stigma or external discrimination can shape career trajectories and long-term economic autonomy, often exacerbated by anachronistic structural and legal barriers in some contexts. For example, in many countries, laws have existed or persist that allowed marriages to be annulled due to epilepsy, in addition to unjustified restrictions in other civil and social spheres (1). Indeed, stigma and its consequences also affect young people with epilepsy in marriage (258). A further concern specific to this stage, and one often left out of the stigma literature despite its evident bearing on it, is the use of valproate in adolescent girls and young women, given its recognized teratogenic profile (259). Fear of future pregnancy, and of the possibility of transmitting harm to a child, is a real and frequently reported source of family and personal distress for those on this medication. The burden it imposes falls unevenly: where epilepsy already carries a higher social cost, in disadvantaged socioeconomic or educational settings, the anxiety surrounding reproductive risk compounds a stigma that is already substantial, and access to the counseling and to the alternative treatment options that might mitigate it is often precisely what is most lacking in such settings.

The transition from pediatric to adult healthcare systems also brings new challenges. This change often coincides with greater personal responsibility for managing the condition and can lead to gaps in care, a sense of loss of support, experiences of disengagement, and difficulties adapting to the new system (236, 260, 261). Adult services are often perceived as less suited to the evolving needs of young people, and the evidence underscores the need for structured, multidisciplinary transition programs that address both medical and psychosocial aspects, prepare the young person for self-management, and facilitate a smooth transfer of care. Taken together, stigma and institutional barriers at this stage can significantly hinder the achievement of economic and social independence (262). As can be seen, stigma remains prevalent in society today, and although the situation is naturally better than in past centuries, it continues to affect the development of children and young people, with consequences that can last for the rest of their lives.

## 6 Toward educational and social inclusion: integrated and collaborative strategies

The preceding sections have argued that stigma is not, at its core, a problem of individual ignorance or misinformation. It is a structural phenomenon, generated by the interaction of power asymmetries, religious and cultural frameworks, and educational exclusion—three factors that recur, with notable consistency, across the entire history traced here. This has a direct consequence for the design of inclusion strategies: interventions aimed exclusively at correcting individual misconceptions, however well-designed, address the symptom rather than the condition. They are more likely to produce temporary improvements in knowledge scores than durable changes in social behavior. The most effective strategies, as the evidence reviewed in this section suggests, share a common feature—they engage with the structural dimensions of stigma, not just its surface manifestations. This is the analytical logic that organizes what follows. Each subsection addresses one of the major domains in which stigma operates against children and young people with epilepsy—education, clinical and psychosocial care, the digital environment, and cross-sectoral collaboration—with attention not only to what interventions exist, but to why some are more likely to produce lasting change than others. The criterion is always the same: does the intervention address the cultural narrative, the institutional practice, or the power relation that generates stigma in that domain? If it does, the evidence tends to support it. If it does not, the evidence tends to show modest, fragile, or context-dependent effects. Given the persistence of the stigma associated with epilepsy, it is necessary to adopt a comprehensive and multidisciplinary approach that coordinates evidence-based interventions across the various settings where it manifests (204, 262–265). Effective mitigation of stigma requires coordinated action across educational, family, social, religious, and healthcare settings, making critical and effective use of available tools and services, and fostering collaboration among stakeholders.

### 6.1 Education and awareness in key settings: school, family, and community

Since perceptions are formed and reinforced through everyday social interaction, educational and awareness-raising strategies are indispensable. In the educational setting, both explicit and implicit biases have to be addressed. Teacher training on epilepsy—which includes recognizing different types of seizures, first aid, educational needs, and debunking myths—improves school staff's knowledge and confidence and fosters more inclusive environments (147, 233, 266). In parallel, educational programs targeting students have been shown to improve knowledge and attitudes toward epilepsy, especially when integrated into structured, age-appropriate interventions (234, 267, 268). In the family setting, interventions should include thorough training on epilepsy, its management, and its psychosocial implications, as well as emotional support and communication strategies adapted to the cultural context. Available evidence shows that communication, coping, and family resilience significantly influence the psychosocial adjustment of children and adolescents with epilepsy (269, 270). In the community, meanwhile, reducing stigma requires culturally sensitive interventions that involve community leaders and explicitly address misconceptions regarding contagion, possession, or supernatural causes. Available reviews show that community campaigns and awareness-raising interventions can improve knowledge and attitudes, although the quality and scalability of the evidence remain uneven (204, 264, 271). In settings where supernatural beliefs or non-biomedical explanations persist, the cultural adaptation of interventions is particularly important (272, 273).

Given its importance, the religious context demands further study, particularly regarding coping strategies (274, 275). There are sensitivities and influences within families and communities where religion plays a significant role. Involving local religious leaders in the processes of helping and supporting children and young people with epilepsy can be decisive in many cases. At times, the strength of faith proves to be very positive. At the same time, in certain settings governed by fanaticism or superstition, healthcare professionals, teachers, and social workers must be properly trained to engage with family members so that they can rationally understand the condition. It must be borne in mind that the social stigma surrounding epilepsy is a powerful force, as history has taught us.

### 6.2 Healthcare integration and youth-centered communication

Epilepsy care cannot be separated from a person's life context; therefore, it requires effective integration with other support systems. Coordinated, interdisciplinary care involves collaboration among neurology, pediatrics, mental health, schools, social work, and primary care. Recent evidence on integrated and comprehensive care for epilepsy highlights that the most promising models incorporate care coordination, communication between services, and an approach centered on the person and their social context (265, 276). In the school setting, training in school nursing and the implementation of individualized seizure action plans can improve safety, return to the classroom, and the quality of support received by students with epilepsy (277–281). In general, it has been shown that knowledge about epilepsy is significantly greater in special schools than in mainstream ones (226); such knowledge is needed if strategies are to address the impact of epilepsy on learning and behavior directly, and if adequate resources are to be available when difficulties arise.

The transition to adult care is a particularly vulnerable period and requires structured programs that prevent care gaps and promote progressive self-management. Available reviews show that advance planning, multidisciplinary involvement, and specific preparation of the young person and their family are central components of a quality transition, although evidence on specific models remains limited (236, 260, 261, 282). In this regard, youth-centered communication matters no less. Moving beyond paternalistic models and promoting clear, developmentally appropriate communication with active patient and family involvement fosters understanding of the diagnosis, treatment adherence, and self-management. In pediatric and adolescent epilepsy, the literature emphasizes the importance of combining education, psychosocial support, and shared decision-making in care and transition processes (260, 261, 269).

### 6.3 The digital environment: combating misinformation and promoting safety

The digital age represents a new cultural phenomenon in the context of the historical evolution we have analyzed. Although we lack prior experience in this regard, there is no doubt that it is now essential to implement specific strategies to counter its risks and capitalize on its opportunities. Misinformation about epilepsy, and about first aid during seizures, circulates widely on social media and other platforms and has to be actively countered by promoting reliable, evidence-based sources and accessible formats. Recent studies show that popular content on platforms such as TikTok may include incomplete or incorrect information about first aid during epileptic seizures, reinforcing the need for digital literacy and the active presence of rigorous content (283). This is a telling example of the risks these networks pose today, risks that extend to both digital and traditional media, as well as to popular artificial intelligence systems—the chatbots commonly used by the public, which remain imprecise and whose misinterpretations can be highly dangerous.

However, digital tools can also be used to improve access to information, therapeutic education, monitoring, and self-management, especially when integrated into broader care models and directly supervised by professionals. Telemedicine and self-care applications have shown potential for reducing access barriers and supporting the daily management of epilepsy, although their usefulness depends on the quality of design, accessibility, and users' digital literacy—meaning there are still many processes to improve and challenges to overcome (163, 284–287). Finally, digital accessibility and safety must be secured for people with photosensitive epilepsy. International evidence and recommendations underscore the need to reduce exposure to visual content with flashing lights or risky patterns and to promote more consistent safety standards in digital and audiovisual environments (182, 288).

### 6.4 The need for cross-sectoral collaboration

Given that stigma is a multifaceted phenomenon with deep-rooted causes, no single strategy is sufficient. Regarding the factors we have focused on, sustained reduction of stigma can only be achieved through intersectoral collaboration among the healthcare and education systems, policymakers, the media, patient organizations, religious entities, and community stakeholders. Reviews and experiences regarding interventions addressing stigma and integrated care agree that coordination across sectors improves message consistency, facilitates inclusion, and increases the likelihood of translating interventions into real-world practice (264, 265, 289–291). Within this framework, there remains a need to strengthen research on the sociocultural manifestations of stigma, barriers to inclusion, and the effectiveness of interventions across contexts—particularly in the stages we have focused on: childhood, adolescence, and youth. There is also a need to promote public policies that eliminate discriminatory provisions, ensure effective protections in education and employment, and fund specific support and inclusion programs (1, 265). Only through coordinated, sustained, and evidence-based efforts that integrate medical, educational, social, and political perspectives can we move toward lasting social inclusion for people with epilepsy (204, 264).

The strategies proposed here carry their own limitations, and these should be stated. A first limitation concerns the source base of the historical analysis itself: the primary and secondary literature consulted weighs more heavily toward Mediterranean, European, and North American traditions than toward Chinese, Indian, and African ones, a consequence of the relative concentration of accessible scholarship in Western academic databases, and this asymmetry should be borne in mind when extending the conclusions of this study to non-Western contexts plainly. The cultural, religious, and socioeconomic heterogeneity of stigma means that any intervention must be carefully adapted to its context; what functions in one setting may be irrelevant or actively counterproductive in another, because the social meanings attached to epilepsy, and the conditions under which care is sought and accessed, vary considerably across regions and healthcare systems. The evidence base for anti-stigma interventions, while growing, remains methodologically uneven—inconsistent in design, often lacking validated outcome measures, and rarely built around robust comparative data (264). Structural problems compound the picture: fragmented care systems, insufficient and unevenly distributed funding, gaps in specialized services, and persistent deficits in treatment availability impose constraints that no educational or awareness-based program can resolve on its own. In the digital sphere, unresolved questions of accessibility, digital literacy, and the variable quality of online health information risk widening existing inequalities rather than narrowing them (284, 292). Cross-sectoral collaboration, for all its demonstrated promise, runs into the practical difficulties that attend any sustained cooperation among organizations with different priorities, institutional cultures, and resource bases (1, 265, 293). A recent large-scale survey of the general population in Italy, published while this work was in preparation, offers a useful contemporary benchmark for this kind of coordinated effort, finding that stigmatizing attitudes toward epilepsy remain measurable even in a high-income European country with well-developed medical services, and that they correlate with specific, identifiable gaps in public knowledge of the condition—a finding consistent with the structural account of stigma developed throughout this research (294).

## 7 Special section—evidence of epilepsy-related stigma affecting children and young people in the Ancient World: an interdisciplinary analysis of a first-century narrative case (Mark 9:14–27)

As noted in Section 3.1, in this special section we present the development of a case study in which primary sources are used to investigate the characteristics of the stigma associated with epilepsy in the past, based on primary evidence. We certainly have examples of the context in which these frameworks of thought emerged and their subsequent development. If we focus on the Near East, which straddles East and West, we can see evidence that stigma against children and young people was constant throughout this historical period. In the 1st century CE, we find one of the most explicit examples, recorded in the Gospel of Mark (Mk 9:14−27), with one of the clearest descriptions of a child's epileptic seizure in the ancient world, specifically in Mk 9:17b−18a, 20cd: […] Διδάσκαλε, ἤνεγκα τὸν υἱόν μου πρὸς σέ, ἔχοντα πνεῦμα ἄλαλον· καὶ ὅπου ἐὰν αὐτὸν καταλάβῃ ῥήσσει αὐτόν, καὶ ἀϕρίζει καὶ τρίζει τοὺς ὀδόντας καὶ ξηραίνεται· […] συνεσπάραξεν αὐτόν, καὶ πεσὼν ἐπὶ τῆς γῆς ἐκυλίετο ἀϕρίζων. This text, in Koine Greek, could be translated as: “Teacher, I brought my son to you; he has a mute spirit, and wherever it seizes him it throws him down, and he foams and grinds his teeth and becomes rigid. […] it convulsed him, and, falling to the ground, he kept rolling about, foaming.” As we can see, the description most likely corresponds to generalized tonic-clonic seizures, which demonstrates remarkable semiological precision in a text from the 1st century CE. The lexical structure shows that the author used common terms from Koine Greek, where καταλαμβάνω retains its basic meaning of “to seize, to lay hold of,” used of what supervenes suddenly and without warning; ῥήγνυμι in its sense of “to throw” or “to knock down” violently, as it also appears in Luke 9:42—the parallels to this passage are Luke 9:37–43 and Matthew 17:14–18—although the more common meaning is “to break” or “to shatter,” the lexica recognizing that here the form has converged with ῥάσσω, “to dash down,” which is the sense the context requires; the verbs of physical manifestation (ἀϕρίζω, τρίζω) with their direct meanings of “to foam” and “to grind”; and the use of the verb ξηραίνω, which, although in its primary sense could be translated as “to dry up” or “to wither”—as we find in Mt 21:19, Mk 11:20, or Jn 15:6—can also mean, depending on the context “to faint/to give out” or “to stiffen, to grow rigid,” which is how we render it here, describing the tonic rigidity of the seizure rather than paralysis in the strict clinical sense—a nuance worth preserving, since the cognate adjective ξηρός is used by the same author of the man's withered hand in Mk 3:3, a related but formally distinct usage—all of which suggests a descriptive narrative of a known phenomenon such as epileptic seizures (85, 295).

Several findings support this view. For example, it is striking that the verb ἀϕρίζω, “to foam” (a denominative in -ίζω on the noun ἀϕρός), occurs in the whole New Testament only in this pericope, twice: ἀϕρίζει, 3rd person singular present active indicative; and ἀϕρίζων, present active participle, nominative singular masculine (the cognate noun ἀϕρός, “foam,” occurs in Luke 9:39). Or even more evident in the hapax legomenon τρίζω, “to grind,” whose sole New Testament occurrence is recorded here (as τρίζει, 3rd person singular, present active indicative), to denote the bruxism resulting from convulsions, which can even damage the temporomandibular joints. The fact that this is a New Testament hapax legomenon underscores the evidence that Mark employs such specific and concrete terms to describe, as accurately as possible, the manifestations characteristic of an epileptic seizure. Its weight lies, however, less in its rarity than in what it displaces: the Septuagint and the rest of the New Testament have a fixed idiom for gnashing the teeth (βρύχω τοὺς ὀδόντας; βρυγμὸς τῶν ὀδόντων), usually with a moral or emotional referent, which Mark declines in favor of a verb whose register is acoustic—an indication that he records a physical sound rather than a scriptural topos. In the passage, different words describe several of the phases of the crisis: the form καταλάβῃ refers to the recurrent onset of the attack, to the moment at which it “seizes” the boy; ῥήσσει indicates violent casting down; ἀϕρίζει and τρίζει refer to oral manifestations; ξηραίνεται may describe rigidity; πεσών and ἐκυλίετο belong to the active episode—denoting loss of consciousness and uncoordinated movements—whereas the death-like condition described subsequently in Mk 9:26 provides a possible indication of a postictal state. Verses 21–22 add an age of onset (ἐκ παιδιόθεν, “from childhood”), a frequency (πολλάκις), and dangerous episodes involving fire and water—burns and submersion remain today among the commonest seizure-related injuries in children (296, 297); the father's closing words, ἵνα ἀπολέσῃ αὐτόν, “in order to destroy him,” show accident being reinterpreted as deliberate assault. However, some of these terms and others like them appear in classical literature and, of course, in the medical literature of the period, in authors such as Aretaeus of Cappadocia (298), who is generally dated to between the first and second centuries CE—although, to imitate the classical style of Hippocrates, he wrote in archaizing Ionic rather than Koine.

All of the above is even more relevant when we consider that the characteristics of Mark's Greek, unlike those of other New Testament authors—such as Luke or the author of the Epistle to the Hebrews—do not exactly stand out for their quality (299). We would describe it as a popular, functional, and direct form of Koine Greek, sometimes characterized as rough and unpolished (300, 301), although, in our case, we should view this with some nuance. His style is characterized by parataxis and the frequent use of the historical present to lend liveliness to the narrative; furthermore, his vocabulary is relatively limited. In any case, he is not a native Greek speaker of high cultural standing. The ancient ecclesiastical tradition, dating back to Papias of Hierapolis, cited in the fourth century by Eusebius of Caesarea in his Ἐκκλησιαστικὴ ἱστορία, Book III, Chapter 39, Section 15 (302), identifies the author with John Mark, a first-generation Jewish-Christian figure associated with Peter in Jerusalem and Rome—his use of Latinisms such as κεντυρίων in 15:39 suggests a Roman audience—and it is consistent with the linguistic profile evident in the Gospel (303). That is, someone functionally competent in Koine Greek but whose linguistic and conceptual matrix would remain essentially Semitic (304–306). This would suggest a probable Aramaic substrate as a reflection of the bilingual environment of the Southern Levant in the first century (307–312), and it is consistent with the Semiticisms present in this Gospel—along with the use of impersonal constructions, the preference for coordination over subordination, and the presence of transliterated Aramaicisms, such as ταλιθα κουμ in 5:41. However, taking into account all these specific characteristics in the author's syntax and lexicon, which may have limited the precision of the description, they did not prevent him from being thorough and precise in his description of an epileptic seizure, employing terms that were undoubtedly widespread in the Koine Greek of his time and which, as noted, were even adopted by other authors in the nosological context.

Furthermore, if we focus on the critical apparatus and the variants of the pericope under analysis, based on modern critical editions of the Greek New Testament, such as the Nestle-Aland, 27th and 28th eds. (313, 314), or The Greek New Testament, 5th ed. (315), we can see that there are no significant discrepancies among the witnesses. To give one example, the variant at Mk 9:20 allows us to outline the development of the interpretation and shows how little interpretive discrepancy the passage admits. We can focus on the use of the verb συσπαράσσω (as συνεσπάραξεν, 3rd person singular, active aorist indicative), in the first variant, which could be translated as “to convulse,” as we have done, or “to tear.” It would refer to a violent convulsion, a spasmodic tremor, or a tear caused by a convulsive movement. In the New Testament, this verb appears only in this verse, Mark 9:20, and in its parallel in Luke 9:42—Luke presumably took it from Mark. This is the variant supported by a compact but weighty cluster of early witnesses—Codex Sinaiticus (א), Codex Vaticanus (B), Codex Ephraemi Rescriptus (C), Codex Regius (L), Codex Sangallensis (Δ) and minuscule 33—and internal evidence may favor it, since Mark uses the simplex elsewhere (1:26; 9:26)—as NA28's own marginal apparatus signals—so that the compound is the more difficult reading and the simplex the likelier scribal substitution, and it is probably what the author of the Gospel of Mark wrote. This is how modern critical editions of the Greek New Testament—such as those mentioned and also the SBL edition (316)—handle it, printing συνεσπάραξεν in their main text. Also of interest is the variant found in Papyrus 45 (𝔓^45^, 3rd c.), Codex Alexandrinus (A), Codex Washingtonianus (W), and other uncial codices and minuscule families, which uses the verb σπαράσσω (with its aorist form ἐσπάραξεν). In this case, we could also translate this word as “he convulsed him” or “he tore him apart,” conveying the same meaning as the first variant. Everything suggests, therefore, that the evangelist was describing an epileptic seizure and was very precise in the terms he used. Moreover, if we start from the fundamental premises regarding the genesis of the New Testament texts and the transmission processes that occurred from oral to written tradition (317–319), we could argue that the text's author was fully aware of what epileptic seizures were like. Most likely, he had witnessed them firsthand at some point, and when he heard the story of the demon-possessed boy whom Jesus heals, he set out, in detail and in a highly descriptive manner, how someone who, in his mind, was possessed would behave and what symptoms they would exhibit. Alternatively, perhaps his source did so, likely based on a historical event, which, for our purposes, is not the most relevant point: it is more important to know that, at that time, epilepsy was associated with a supernatural phenomenon of evil possession for which an exorcism was necessary.

In this sense, the passage is not only important to us from a modern epileptological perspective but also provides a characterization of ictal motor phenomena, autonomic manifestations, and postictal alterations, which remain descriptively recognizable to contemporary neurology. However, modern diagnosis rests on criteria of an entirely different nature. In reality, beyond describing an epileptic seizure with relative precision—which is in itself quite remarkable—what is of greatest significance in our case is how it shows us in detail the context in which the stigma is present. If we analyze this perspective, we can see how the author alludes to the fact that the cause of this child's convulsions is a mute spirit (πνεῦμα ἄλαλον). The epithet names the spirit from the deficit it is held to cause, and can be interpreted precisely because the boy utters nothing during the episode, unlike other phenomena in which the affected person might utter screams or curses—as in Mark 1:23–26/Luke 4:33–35 or Mark 5:1–13 and their parallels, which from a clinical perspective could be related to acute psychotic episodes, schizophrenia with agitation, dissociative identity disorder, Tourette syndrome, or even a focal seizure with perseverative verbal automatism, among others—all of which underscores that we are dealing with a person experiencing generalized tonic-clonic seizures. What the text emphasizes is the absence of articulate speech, not the absence of sound: verse 26 attributes a cry (κράξας) to the departing spirit; phenomenologically, this may evoke the ictal cry characteristic of this clinical pattern.

Understanding it as a possession would have been a given at that time, and that supposed origin frightened people. Those who suffered such episodes were stigmatized. The evangelist is very descriptive of this, painting a vivid scene in which a crowd gathers around the disciples while scribes (γραμματεῖς) dispute with them (9:14). The presence of the scribes, teachers of the Law whom Mark often associates with the chief priests (8:31; 10:33; 11:18; 11:27), reinforces the supernatural significance and importance of the event and shows that what is at issue is not merely the cure but a question of authority, disputed over the child's body. It must be borne in mind that, in the context of Second Temple Judaism, for the more traditional sects—such as the Sadducees, who denied the resurrection (Mk 12:18; Acts 23:8)—divine retribution for good and evil was still considered to depend on our actions: those who fulfill divine commandments enjoy happiness, and those who do not suffer. And this was in this life. Sheol—שְׁאוֹל, whose etymological origin has been related by some to the root שׁאל (sha'al), meaning “to ask” or “to demand,” and by others to שׁאה, “to be desolate,” and whose interpretation is still under debate—equivalent to the Greek Hades, was a place beyond the grave common to both the righteous and the wicked. Therefore, the theological controversies likely reflected in the passage—which the writer may be highlighting by mentioning the scribes—tell us of these new movements and communities, such as that of Jesus, in which influences from external schools of thought led to the consideration of a post-mortem divine reward, with a final judgment that would result in two distinct destinies: resurrection and eternal life in the Kingdom of God for the righteous—especially those devoted to the most disadvantaged—and Gehenna (γέεννα, from הִנֹּםגֵּי, the Valley of Hinnom, where the Hebrew Bible places the immolation of children to the god Molech [2 Kgs 16:3; 21:6; 23:10; Jer 7:31; 32:35], a practice condemned as abhorrent and alien, very different from the idea of Sheol) for the wicked (320). But in this context, various currents of thought would understand epilepsy in children as divine punishment for the sins of their parents (cf. Jn 9:2).

It is also possible to identify other complementary interpretations. In the case at hand, in Mark, the mechanism of stigmatization is the presence of an evil force within the child's body. In the parallel passage in Matthew (17:15), by contrast, the author used a colloquial term for those who have epilepsy, σεληνιάζομαι—medical writers commonly used ἐπιλη*Ppsi*ία, from ἐπιλαμβάνω, “to lay hold upon,” the same root as the καταλαμβάνω of Mark's account. The verb alludes to a belief—likely with antecedents in Mesopotamian etiology (321)—according to which the cause of these possessions was the influence of the moon; it is the Greek semantic analog of the Latin lunaticus and of a long European tradition that persisted for centuries. Matthew nonetheless retains the demon (17:18), so that the two etiologies are superimposed rather than substituted, and in any case it was understood as a supernatural phenomenon.

It should be repeated that the evangelists operated within a demonological etiological framework to explain illnesses that we today classify as neurological and psychiatric. Their goal was not nosological precision, but rather to bear witness to the thaumaturgical power of Jesus over evil, whatever its manifestation, and, at the same time—as we discuss in Section 3.3—to highlight Jesus' criticism of the crowd for stigmatizing the boy. In general, the text analyzed reflects a conceptual framework in which the physical manifestations of epilepsy were understood as the visible effects of possession or spiritual influence, which could affect and even mark children from birth or earliest childhood.

## 8 Conclusions

Seven sections of historical and contemporary analysis converge on a picture that is, in some respects, more complex than prior accounts have suggested. The stigma associated with epilepsy in children and young people has not simply persisted alongside scientific progress; it has, at each successive historical moment, adapted to whatever conceptual framework happened to be available—supernatural, moral, psychiatric, genetic, or ideological (38). That adaptability is, perhaps, the most significant finding of this study, and the one with the most direct bearing on contemporary intervention.

A first conclusion, and one that deserves more attention than it has received in the literature, concerns what the present analysis reveals about the structure of epileptic stigma. Across cultures as distant as Mesopotamia, classical Rome, medieval Europe, and early modern religious movements, the identical cluster of phenomena—seizures, loss of consciousness, involuntary motor manifestations—was assigned radically different social meanings depending on the position of the affected individual within prevailing hierarchies of power, wealth, and religious authority. Among the poor, the marginal, and the socially excluded, such manifestations were consistently read as signs of demonic possession, divine punishment for familial sin, or inherited moral corruption. Among those who occupied positions of political power, economic privilege, or spiritual eminence, the same or analogous phenomena were recast—often by the very same cultural logic—as marks of divine election, prophetic capacity, or charismatic predestination. Julius Caesar, in the Roman sources, is perhaps the most historically prominent case; similar patterns can be traced through the figures associated with mystical and religious authority in multiple traditions. This status-contingent inversion of stigmatic valence—what one might call the differential social coding of epileptic symptomatology according to the subject's location in structures of power—is a structural feature of the social construction of epilepsy-related stigma, recurring with notable consistency across historical periods. The implications are not merely antiquarian. They speak directly to the persistence, in contemporary societies, of differential stigmatization along socioeconomic lines, where epilepsy continues to carry a disproportionate psychosocial burden for children and young people in conditions of poverty, geographic marginalization, and limited access to education and medical care (90). This axis of analysis—the modulation of stigma by power, economic context, and religious status—has been underexplored in existing scholarship, which has tended to treat stigma as a relatively uniform social phenomenon.

A second conclusion concerns method. The interdisciplinary analysis presented in Section 7—which brings together New Testament Greek philology, the direct analysis of original-language sources, systematic textual criticism based on the major critical editions of the Greek New Testament, historical-critical biblical scholarship, the social history of Second Temple Judaism, and contemporary epileptological semiology—demonstrates what becomes analytically visible when the disciplinary conventions of a single field are set aside. The outcome includes, among other things, a phase-by-phase correlation between the Greek text of Mark 9:14–27 and modern ictal phenomenology—tonic, clonic, and postictal phases—that allows one of the oldest surviving descriptions of a childhood epileptic seizure to be evaluated in terms of contemporary diagnostic criteria. The textual analysis is grounded in the manuscript tradition: variant readings from the Codex Sinaiticus, the Codex Vaticanus, the Codex Alexandrinus, and others are examined, and the principles of internal evidence are applied to establish the most probable original reading (322). But beyond the specific findings, the section makes a methodological argument: that the historical study of epilepsy-related stigma can draw productively on methods from the humanities—philological, literary, theological, historical—that are rarely brought to bear on questions in the health and social sciences. The specific convergence attempted here is, to our knowledge, an uncommon one in the literature on stigma and epilepsy.

Beyond these specific contributions, the present analysis confirms what has long been suspected but not always stated directly: scientific advance does not automatically erode social stigma, and may, in certain historical moments, simply furnish it with new vocabulary (133). The eugenics movement of the late nineteenth and early twentieth centuries—which persisted in various institutional and geographical forms well into the second half of the twentieth century—warrants separate comment, since it undermines a narrative that the existing literature has sometimes allowed to remain unquestioned. That narrative maintains, broadly, that scientific progress is the natural enemy of stigma, and that the displacement of supernatural or religious explanations by neurological ones constitutes advance both moral and intellectual. The eugenic case holds the contrary. A condition classified with increasing clinical precision, documented in its hereditary patterns, and discussed in the authoritative language of medicine and public health simultaneously became the object of coercive sterilization legislation in more than two dozen American states (323) and, in its most extreme form, provided the pseudoscientific basis for systematic extermination under National Socialism (324). The stigma associated with epilepsy during this period was no less virulent for having shed its religious framing; on the contrary, backed as it was by institutional authority, it became harder to challenge. Religious stigma can be answered with a theological argument; scientific stigma, by contrast, cloaks itself in a language purportedly grounded in science—though at its core it serves political or ideological interests—that seeks to foreclose any avenue of reply. This observation has direct implications for the present: it cautions against the comfortable assumption that greater neurological knowledge will, of itself, produce more tolerant social attitudes. Knowledge and attitude run, on many occasions, along separate paths, and the history of epilepsy makes this clear at several points—none more starkly than this one.

For children and young people specifically, the accumulation of stigmatic injury across the life course—in identity formation, educational trajectory, social relationships, and future prospects—demands coordinated intervention across clinical, educational, social, and policy domains. The evidence examined here suggests, consistently, that the most durable interventions are those that engage with the cultural narratives, religious frameworks, and educational practices that sustain stigma, rather than treating it as a problem of simple misinformation. The digital environment adds dimensions—cyberbullying, algorithmic amplification of stigmatizing content, specific risks for those with photosensitive epilepsy—that are without historical precedent and for which the field lacks fully developed responses. Research will need to continue examining how historical patterns of stigmatization manifest in contemporary contexts, with particular attention to intersectional factors—socioeconomic status, gender, ethnicity, and geographic location—that compound discrimination (325). The present study has shown that stigma is not a monolithic social phenomenon but a shifting, context-dependent one, whose valence is shaped at every point by relations of power. Taking that finding seriously is, we believe, the starting point for any serious effort at mitigation.
